# The polyamine inhibitor SAM486A increases the efficacy of adagrasib in non-small cell lung cancer cells harboring KRAS^G12C^ mutation

**DOI:** 10.1186/s40659-026-00679-w

**Published:** 2026-02-19

**Authors:** Antonia Martin-Martin, Carina Chipón, Constanza Guzman-Kunstmann, Sergio Guzman-Kunstmann, Neudo Buelvas, Claudio Henríquez, Francisca Vidal, Franz Villarroel-Espindola, Trista K. Hinz, Lynn E. Heasley, Rodrigo A. López-Muñoz

**Affiliations:** 1 Instituto de Farmacología y Morfofisiología, Facultad de Ciencias Veterinarias, Universidad Austral de Chile, PO. 5090000, Valdivia, Chile; 2 School of Dental Medicine, University of Colorado Anschutz Medical Campus, Aurora, CO 80045 USA; 3 Unidad Medicina Traslacional, Instituto Oncológico Fundación Arturo López Pérez, Santiago, Chile

**Keywords:** Non-small cell lung cancer, KRAS inhibitors, Polyamines, Drug combinations

## Abstract

**Background:**

Non-small cell lung cancer (NSCLC) accounts for most lung cancer cases and poses major challenges due to late-stage diagnosis and limited options. A substantial subset of NSCLC harbors KRAS mutations, most commonly at codon 12. Although KRAS^G12C^ inhibitors show clinical activity, their efficacy is frequently limited by acquired resistance. Polyamines (putrescine, spermidine, and spermine) regulate key cellular processes and are dysregulated during tumorigenesis. S-adenosylmethionine decarboxylase 1 (AMD1) inhibitors such as SAM486A reduce tumor cell proliferation and migration. Thus, we evaluated the combination of the KRAS^G12C^ inhibitor adagrasib with SAM486A in KRAS^G12C^-mutant NSCLC, in vitro and in vivo.

**Methods:**

In vitro assays included viability (MTT), clonogenic, BrdU incorporation, and Western blot analyses across four NSCLC cell lines. Drug–drug interactions were quantified using Combenefit software. In vivo efficacy was tested in C57BL/6 mice using an orthotopic model with LLC46 (KRAS^G12C^/NRAS^KO^) cells and a metastatic model with LL2 (KRAS^G12C^/NRAS^Q61H^) cells. Tumor growth was monitored by µCT or caliper measurements, and immunohistochemistry (PCNA) was used to assess proliferation.

**Results:**

In vitro, adagrasib reduced AMD1 levels, and SAM486A synergistically enhanced its antiproliferative effects, particularly in KRAS^G12C^-mutant cell lines, with minimal effects on KRAS–wild-type cells. In an orthotopic mouse model using a KRAS^G12C^/NRAS^KO^ NSCLC line, the combination provided minimal additional benefit over adagrasib monotherapy. In a metastatic model, however, the combination reduced tumor size and PCNA staining more than either monotherapy.

**Conclusions:**

Our results suggest that targeting polyamine metabolism with SAM486A enhances the efficacy of KRAS^G12C^ inhibitors and may mitigate resistance. This combination represents a promising therapeutic approach for KRAS^G12C^-mutant NSCLC and warrants further clinical investigation.

## Introduction

Lung cancer is the leading cause of cancer-related mortality worldwide, with 2.4 million new cases annually and 1.8 million deaths across both sexes [9]. Non-small cell lung cancer (NSCLC) constitutes ~ 85% of lung cancer cases [6]. Mutations in the KRAS oncogene occur in 20–30% of NSCLC patients, predominantly in adenocarcinomas, with a higher prevalence among smokers (30%) compared to non-smokers (10%) [1]. These mutations are associated with poor prognosis and increased risk of recurrence [7].

Among the KRAS variants, three substitutions at codon 12 predominate in NSCLC, resulting in amino acid changes from glycine to cysteine (KRAS^G12C^, ~ 40%), valine (KRAS^G12V^, ~ 22%), or aspartate (KRAS^G12D^, ~ 16%) [19]. Notably, KRAS^G12C^ and KRAS^G12V^ are particularly linked to worse survival [23]. Adagrasib is a selective covalent inhibitor of KRAS^G12C^ that binds irreversibly to the cysteine residue at codon 12, maintaining the protein in its inactive GDP-bound “OFF” state and thereby blocking downstream KRAS-dependent signaling [20, 26]. Owing to its favorable pharmacokinetic properties, including a prolonged half-life and central nervous system penetration, adagrasib has been evaluated in patients with advanced NSCLC harboring KRAS^G12C^ mutations and brain metastases, demonstrating intracranial activity in the KRYSTAL-1 trial [24]. Based on these results, adagrasib received accelerated FDA approval in December 2022 for the treatment of adult patients with KRAS^G12C^-mutated locally advanced or metastatic NSCLC who had previously received at least one systemic therapy [14].

Despite the groundbreaking discovery of selective KRAS^G12C^ inhibitors, monotherapies targeting KRAS^G12C^ have been associated with acquired resistance in various tumor models. Such resistance results in the upregulation of alternative survival pathways, both in experimental systems and in patients [17, 29]. Mechanisms of resistance to adagrasib monotherapy have been investigated in patients with KRAS^G12C^-mutant cancers (lung, colon, and appendix), and can be broadly categorized into three groups: (i) secondary mutations or amplifications in KRAS that prevent adagrasib binding; (ii) oncogenic alterations that activate alternative RTK–RAS signaling pathways without directly affecting KRAS; and (iii) phenotypic transformation from lung adenocarcinoma to squamous cell carcinoma [3]. These findings highlight the need to deepen our understanding of the metabolic implications of KRAS inhibition in order to identify combination therapies that maximize inhibitor efficacy while preventing resistance.

Natural polyamines (putrescine, spermidine, and spermine) are small cations that play essential roles in cell proliferation and function, and are often dysregulated in hyperproliferative disorders such as cancer [12, 22]. Elevated polyamine levels in plasma and urine are associated with poor prognosis in cancer patients, including those with NSCLC [25]. One of the rate-limiting steps in polyamine biosynthesis is the decarboxylation of S-adenosyl-L-methionine by S-adenosyl-L-methionine decarboxylase (AMD1), which is essential for the synthesis of spermidine and spermine [10]. AMD1 has been implicated in multiple cancers, including prostate, breast, and gastric malignancies, and its activity has been linked to KRAS signaling through the PI3K/AKT/mTOR pathway [42, 45].

SAM486A is the best-characterized inhibitor of AMD1 [35], and has shown promise as a cancer therapeutic due to its ability to reduce tumor cell proliferation and migration [11]. SAM486A has previously been tested as monotherapy in several phase I and II clinical trials involving patients with solid tumors and hematologic malignancies [31, 34, 38]. These early studies identified dose-limiting neutropenia as the main hematologic adverse event, along with mild to moderate non-hematologic toxicities such as nausea, fatigue, and anorexia. More recently, preclinical work in NSCLC models demonstrated that SAM486A exerts antiproliferative and antimigratory effects in NSCLC, where treatment induced metabolic alterations such as methionine accumulation, cysteine depletion, and reduced spermidine levels, changes that were accompanied by increased global DNA methylation [33].

Therefore, this study aims to evaluate the effect of combining the KRAS^G12C^ inhibitor adagrasib with the polyamine inhibitor SAM486A in NSCLC cells harboring the KRAS^G12C^ mutation, both in vitro and in vivo.

## Materials and methods

### Cell lines and culture method

We utilized the human NSCLC cell lines H1299 (ATCC^®^CRL-5803™), A549 (ATCC^®^CRM-CCL-185™), CALU-1 (ATCC^®^HTB-54™), and H358 (ATCC^®^CRL-5807™). Also, we used the murine cell lines LL2 (Lewis Lung Carcinoma cells, ATCC^®^RL-1642™) and the LL2-derived cell line LLC46, (KRAS^G12C^/NRAS^KO^) generated in the laboratory of Dr. Lynn Heasley (University of Colorado) [37]. Cells were cultured using RPMI 1640 and culture media, supplemented with 10% fetal bovine serum (FBS) and 1% antibiotics (penicillin, 100 U/mL and streptomycin, 100 µg/mL; Biological Industries, Beit HaEmek, Israel). The cells were incubated in a humidified environment at 37 °C with 5% CO_2_. To subculture the cells, we washed them with Phosphate-Buffered Saline (PBS) and added a solution of 0.25% trypsin and 0.03% EDTA (Sartorius, Beit HaEmek, Israel). After incubating the cells for 1 min, we added complete medium, centrifuged the cells for 8 min at 600 × *g*, resuspended the pellet in complete medium, and counted the cells using a Countess II automated cell counter (ThermoFisher Scientific, Waltham, MA, USA). All cell lines were used until passage 20. All cell lines were routinely screened for mycoplasma contamination every 60 days using the Mycoplasma PCR Detection Kit (cat. #G238, abm, Richmond, BC, Canada), according to the manufacturer’s instructions. All experiments were performed using mycoplasma-free cultures.

### Drugs

The KRAS^G12C^ inhibitors adagrasib (CAT# HY-130149/CS-0105265) and sotorasib (CAT# HY-114277/CS-0081316) and the polyamine inhibitor SAM486A (CAT# HY-13746B/CS-0028219) were purchased from MedChem Express^®^, Monmouth Junction, NJ, USA. All drugs were dissolved in dimethyl sulfoxide (DMSO, Merck, Darmstadt, Germany) and assayed at nine concentrations to generate concentration-response curves and combination models. The DMSO concentration in the cultures never exceeded 0.5% v/v.

### In vitro combinations

Cell seeding, serial twofold dilutions of drugs, and viability measurements were performed in 96-well plates. Experiments included at least five concentrations of each drug and all possible combinations arranged in a checkerboard matrix. Drug combinations were analyzed using Combenefit software V2.021, which evaluates interactions in terms of synergy, additivity, or antagonism. The analysis was performed using the Loewe additive model and the SUM_SYN_ANT_WEIGHTED (SSAW) parameter was selected as a global score to summarize combination effects across the concentration-response matrix [15]. At least four replicates per combination were evaluated. Combenefit applied a Student’s t-test to compare viability data, with statistical significance set at a *p*-value < 0.05, to identify differences between observed viability and the theoretical combination model, which assumes no drug interaction. To improve the visualization and interpretation of combinatorial interactions, heatmaps were generated using Python (version 3.11) with the Matplotlib and NumPy libraries. For each cell line, a matrix of synergy scores was plotted using a diverging color scale (jet_r colormap), centered at zero (green), with synergy values up to + 40 (blue) and antagonism down to -40 (red). Mean synergy scores were overlaid in bold at each grid point, and standard deviations were displayed beneath in smaller font using the format: ± value. Axis labels correspond to the tested concentrations of Adagrasib and SAM486A, and were adapted to match the specific design of each matrix used in the experiments.

### Cell viability

NSCLC cell lines were seeded in 96-well plates. After 24 h of acclimatation, cells were exposed to the drugs for 72 h. Subsequently, cell viability was evaluated through incubation with 3-(4,5-dimethylthiazol-2-yl)-2,5-diphenyltetrazolium bromide (MTT) (0.5 mg/mL, Sigma-Aldrich, St Louis, MO, USA) for 4 h. The formed crystals were solubilized through incubation with 100 µL of 10% SDS in 0.01 M hydrochloride overnight at 37 °C. The production of formazan was measured at 579–690 nm in a TECAN Infinity MPRO 200 (Tecan Group, Grödig, Austria).

### Clonogenic assay

Human and murine NSCLC cell lines were seeded in 12-well plates (500 cells/well). Twenty-four hours after seeding (time 0), cells were treated with SAM486A at the indicated concentrations for 72 h. After completion of the treatment period (96 h from initial seeding), the culture medium containing the drug was removed and replaced with fresh drug-free medium. Following drug removal, cells were allowed to grow for additional periods that varied according to the cell line: LL2 and LLC46 cells were cultured for an additional 3 days (total experimental duration of 168 h); CALU-1 cells were cultured for 15 additional days; H358 cells for 10 additional days; and H1299 and A549 cells for 7 additional days. In all cases, fresh medium was replaced every 3 days during the post-treatment growth phase. These different post-treatment incubation times were selected to account for differences in cell line–specific doubling times colony formation rates observed in our laboratory. Subsequently, colonies were fixed in 100% cold methanol for 20 min, washed, and stained with 0.5% crystal violet (CV) solution for 5 min. The plates were air-dried, and colonies were visualized using a Microdirect™ digital microscope at 40× magnification (Celestron, Torrance, CA, USA).

### BrdU proliferation assay

Human NSCLC cell lines were seeded in 96-well plates (10.000 cells/well in the case of H1299 and A549 cells and 15.000 cells/well for H358 and CALU-1) and after overnight acclimatization, the cells were treated with different concentrations of SAM486A for 24 h. The BrdU uptake was measured by using the BrdU Cell Proliferation Assay Kit (CAT# 2752, Merck, Burlington, MA USA), following the manufacturer’s instructions. Briefly, five hours before the end of treatment, BrdU solution was added and incubated with cells at 37 °C with 5% CO_2_. Finally, the cells were fixed, and the incorporation of BrdU was evaluated by ELISA.

### Western blotting

NSCLC cells were seeded in six-well plates (1 × 10^6^ cells per well) and allowed to attach for 24 h, after which cells were exposed to the drugs for 1, 6, 12, or 24 h. Cell lysates were prepared using Pierce™ radioimmunoprecipitation assay (RIPA) buffer (Thermo Fisher Scientific, Rockford, IL, USA) containing 25 mM Tris-hydrochloride (pH 7.6), 150 mM sodium chloride, 1% NP-40, 1% sodium deoxycholate, and 0.1% sodium dodecyl sulfate (SDS), supplemented with Halt™ protease inhibitor cocktail (Thermo Fisher Scientific, Rockford, IL, USA). Proteins were extracted and centrifuged for 15 min at 15,000 *g* at 4 °C. Total protein concentration was determined using the Protein Assay Dye Reagent Concentrate (Bio-Rad Laboratories, Hercules, CA, USA). Subsequently, 80 µg of total proteins were denatured at 95 °C for 5 min and resolved using 15% SDS-polyacrylamide gel electrophoresis at 25 mA. Proteins were electrotransferred to a Protran™ pure nitrocellulose membrane 0.45 mm (PerkinElmer, Waltham, MA, USA) at 450 mA for 1 h. The membrane was blocked using 5% non-fat milk in Tris-buffered saline containing 0.1% Tween 20 (TBST, pH 7.4) for 1 h at room temperature and washed with TBST.

For AMD1 immunoblotting, the membranes were incubated overnight with primary antibodies against AMD1 (CAT# SC-166970, 1:1,000) and vinculin (CAT# sc-73614, 1:10,000) as loading control in 5% non-fat milk in 0.1% TBST at 4 °C. The membranes were washed with 0.1% TBST and incubated for 90 min with a secondary anti-mouse IgG-HRP (CAT# 7076 S, 1:5,000) antibody in 5% milk in 0.1% TBST. The AMD1 and vinculin antibodies were purchased from Santa Cruz Biotechnology (Dallas, TX, USA), and the anti-mouse IgG-HRP was purchased from Cell Signaling Technology (Danvers, MA, USA). Following incubation, the membranes were washed with 0.1% TBST.

For ERK1/2 phosphorylation analysis, the membranes were incubated overnight with primary antibodies against phospho-p44/42 MAPK (ERK1/2) (CAT# 9101 S, 1:1000) and vinculin (CAT# sc-73614, 1:10,000) as loading control in 5% BSA in 0.1% TBST at 4 °C. The membranes were washed with 0.1% TBST and incubated during 90 min with a secondary anti-rabbit immunoglobulin G conjugated to horseradish peroxidase (IgG-HRP) (CAT# 7074 S 1:5000) or anti-mouse IgG-HRP (Cat #7076S, 1:5000) antibody in 5% milk in 0.1% TBST.

Blots were imaged using an Odyssey^®^ Fc Western blot scanner (LI-COR Biosciences, Lincoln, NE, USA). Western blotting quantitation was performed using the Image Studio Lite version 5.0 (LI-COR Biosciences).

### In vivo orthotopic model for tumor growth

8 week old female C57B/6J mice were purchased from Jackson Labs (Bar Harbor, ME) and housed in the animal facilities of the University of Colorado, Anschutz Medical Campus. For tumor cell inoculation, LLC46 (KRAS^G12C^/NRAS^KO^) [37], cells were grown to 75% confluence, harvested, and resuspended in sterile phosphate-buffered saline. Cells were counted and resuspended to a final concentration of 3,125 cells per µL. 40 µL of the cell suspensions were directly injected into the left lung of mice through the ribcage. Mice were randomized into groups to receive diluent control, SAM486A (30 mg/kg; MedChem Express, Monmouth Junction, NJ, USA), adagrasib (30 mg/kg; MedChem Express Monmouth Junction, NJ, USA), or the combination by daily oral gavage until primary experimental endpoints were met. Treatment was initiated following confirmation that at least 75% of mice had measurable primary left lung tumors via µCT scanning (Small-Animal Image-Guided Radiation Therapy shared resource, University of Colorado Cancer Center). µCT scanning was conducted weekly and ITK-SNAP 4.0 software [44] was used to calculate cross-sectional tumor volumes in cubic millimeters.

### In vivo metastatic model for tumor growth

Eight-week-old female C57BL/6 mice were bred and housed at the animal facilities of the Institute of Pharmacology and Morphophysiology, Faculty of Veterinary Sciences, Austral University of Chile. LL2 cells were cultured to 75% confluence, harvested, and resuspended in sterile PBS. Cells were counted and adjusted to a final concentration of 12,500 cells/µL. A total of 40 µL of the cell suspension was injected into the lateral tail vein.

Mice were randomized into treatment groups receiving vehicle control, SAM486A (25 mg/kg; MedChem Express), adagrasib (25 mg/kg; MedChem Express), or the combination therapy. Treatments were administered daily via oral gavage until experimental endpoints were reached. Tumor size was assessed by measuring tumor diameters with a caliper. On day 21, mice were euthanized via intraperitoneal injection of 100 µL of ketamine (10 mg/mL) combined with xylazine (1 mg/mL). Tumors were extracted under sterile conditions and immediately fixed in Bouin’s solution.

### Ethical statement

All animal procedures were conducted in accordance with institutional and national guidelines for the care and use of laboratory animals. The orthotopic lung tumor model study was reviewed and approved by the Institutional Animal Care and Use Committee (IACUC) at the University of Colorado Anschutz Medical Campus. The metastatic model study was approved by the Institutional Committee for the Care and Use of Animals (CICUA) at the Universidad Austral de Chile (Protocol CICUA 391/2020). Mice exhibiting signs of morbidity were euthanized immediately using isoflurane anesthesia followed by cervical dislocation, in compliance with ethical and humane standards.

### Immunohistochemistry

Bouin's-fixed lungs were dewaxed, rehydrated, and blocked for endogenous peroxidase activity. Heat-Induced Epitope Retrieval (HIER) was performed for 20 minutes at 97 °C using EnVision FLEX Target Retrieval Solution, High pH (Dako Omnis) in a PT-module from Thermo Scientific. Slides were incubated with a 1:4,000 dilution of anti-proliferating cell nuclear antigen (PCNA) (CAT# 2586S, Cell Signaling) for 30 minutes. Slides were washed and incubated with Dako K4001 anti-mouse IgG secondary antibodies for 30 minutes. Sections were developed with diaminobenzidine (DAB) substrate plus the DAKO brand Substrate Chromogen System kit. Images were obtained with an Aperio-Versa microscope and the Aperio Cellular IF Algorithm software (Leyca biosystems, Nussloch, Germany).

### PCNA immunohistochemistry analysis

Representative regions of interest (ROIs) were exported from Aperio ImageScope as high-resolution TIFF files and analyzed using QuPath (version 0.6.0) [4]. Images were imported into QuPath, and scale calibration was verified prior to analysis. Tissue regions were manually annotated to define ROIs, and automated cell detection was performed using QuPath’s cell detection algorithm to segment individual nuclei based on intrinsic nuclear contrast. Nuclear segmentation parameters were kept constant across all samples.

DAB signal quantification was performed following internal color deconvolution (H-DAB). For each detected nucleus, the mean optical density (OD) of the DAB signal within the nuclear compartment (Nucleus: DAB OD mean) was calculated. Nuclei were classified as PCNA-positive or PCNA-negative using a fixed DAB OD threshold applied uniformly across all images. The threshold was defined empirically based on the distribution of nuclear DAB OD values and visual inspection of representative control sections, and was not modified between experimental groups.

For each ROI, the percentage of PCNA-positive cells was calculated as the proportion of PCNA-positive nuclei relative to the total number of detected nuclei. In addition, PCNA staining intensity was quantified as the mean nuclear DAB OD considering only PCNA-positive nuclei. All single-cell measurements were exported as tab-delimited text files and aggregated at the ROI level for downstream statistical analysis.

### Statistical analysis

All data are expressed as mean ± standard deviation (SD), unless otherwise indicated. Statistical analyses were performed using GraphPad Prism software (version 10.6.1). For Western blot quantifications evaluating the effect of treatments over time, statistical significance between treated and control conditions was assessed using two-way analysis of variance (ANOVA). For concentration–response experiments (MTT and BrdU assays), statistical differences between cell lines were evaluated by pairwise comparison of dose–response curves using extra sum-of-squares F tests, as implemented in GraphPad Prism. For analyses involving tumor number and diameter, one-way ANOVA followed by Dunnett’s multiple comparisons *post hoc* test was applied. For comparisons of PCNA staining intensity between control and combination-treated groups, an unpaired two-tailed *t* test with Welch’s correction was applied to account for unequal variances between groups. Survival curves in the orthotopic model were generated using the Kaplan–Meier method. Drug–drug interaction analyses were performed using the Loewe additivity model implemented in the Combenefit software (version 2.021) [15] For each combination point, deviations from the theoretical additivity model were statistically evaluated using Student’s t-test, as implemented in the software. In all analyses, a p-value < 0.05 was considered statistically significant.

## Results

### SAM486A exhibits antiproliferative activity and synergizes with the KRAS^G12C^ inhibitor adagrasib in NSCLC cells

**Fig. 1:**
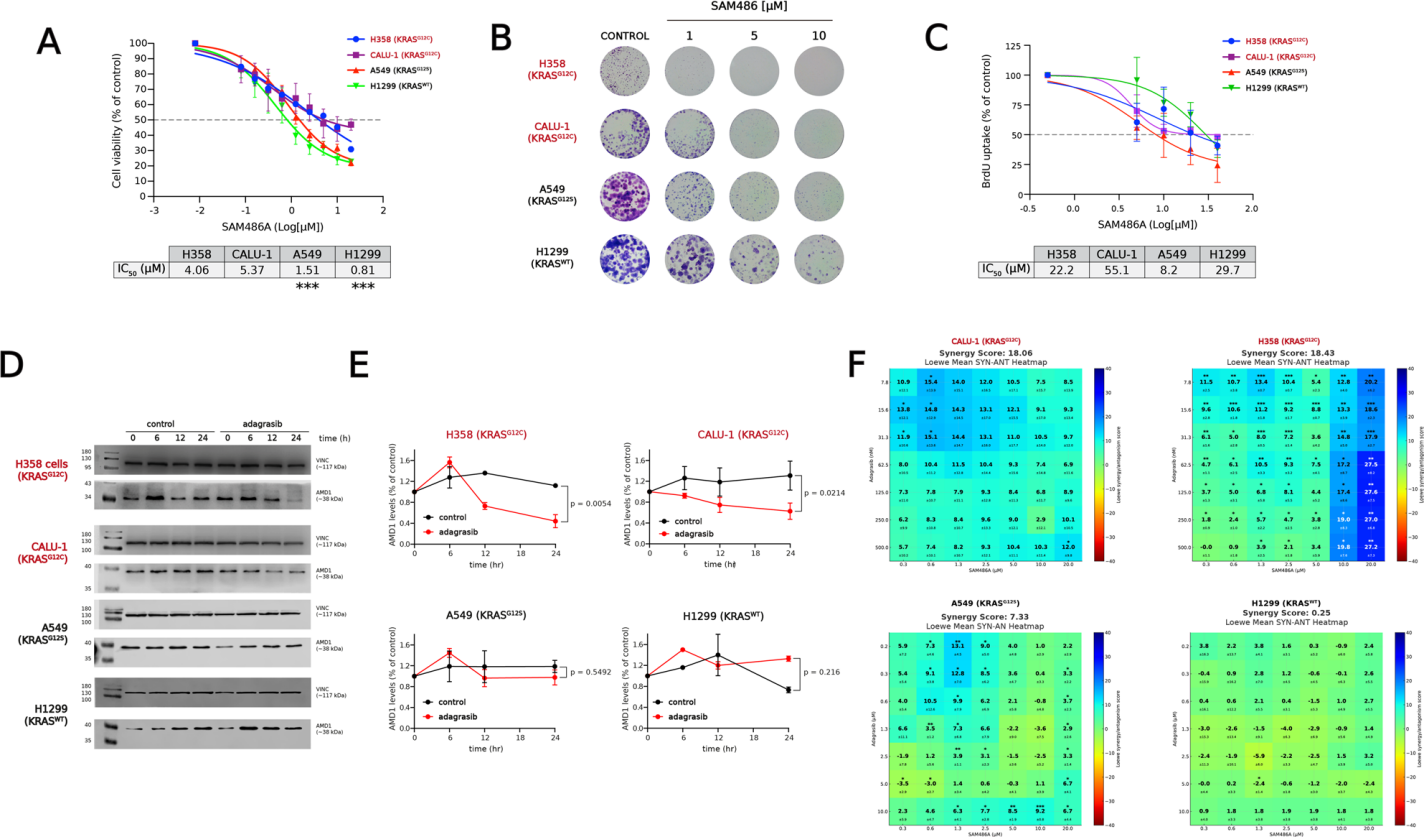
Effect of SAM486A, adagrasib and their combination on NSCLC cells harboring the KRAS ^G12C^ mutation. **A** Dose–response curves of SAM486A in H358 (KRAS^G12C^), CALU-1 (KRAS^G12C^), A549 (KRAS^G12S^), and H1299 (KRAS^WT^) cells assessed by MTT assays after 96 h of treatment. IC₅₀ values are summarized in the table. ***: *p* < 0.001, compared with H358 and CALU-1 cells, as determined by pairwise extra sum-of-squares F tests applied to the concentration–response curves. **B** Representative images of colony formation after 7 days of exposure to increasing concentrations of SAM486A (1–10 µM), stained with crystal violet. **C** Dose–response curves of BrdU uptake in NSCLC cells exposed to SAM486A for 24 h, reflecting proliferation rates. IC₅₀ values are shown in the table. No statistically significant differences in EC₅₀ values were observed between the conditions analyzed. **D** Representative immunoblots of AMD1 protein levels in cells treated with adagrasib (0.5 µM) for 6–24 h. Vinculin was used as a loading control. **E** Quantification of AMD1 protein levels from three independent experiments (mean ± SD). p-values indicate significant differences between treated and control groups, calculated using two-way ANOVA. **F** Synergy maps of SAM486A combined with adagrasib in NSCLC cells. Synergy scores were calculated using Combenefit with the Loewe additivity model. Blue shades indicate synergistic interactions, while red shades indicate antagonism. Data represent three independent experiments performed in duplicate. Asterisks indicate statistical significance relative to the reference model (Loewe) for each combination point, calculated by t-test. *: *p* < 0.05, **: *p* < 0.001, ***: *p* < 0.0001, ****: *p* < 0.00001

First, we evaluated the effect of the AMD1 inhibitor SAM486A on the viability of four NSCLC cell lines (H358, CALU-1, A549, and H1299). Cells harboring the KRAS^G12C^ mutation (H358 and CALU-1) exhibited significantly lower sensitivity to SAM486A than KRAS non-G12C cell lines, as determined by pairwise statistical comparison of concentration–response curves (Fig. 1A). The IC₅₀ values for H358, CALU-1, A549, and H1299 were 4.06, 5.37, 1.51, and 0.81 µM, respectively (Fig. 1A). However, this effect on cell viability did not appear to correlate with proliferation assays. Colony formation assays (Fig. 1B) indicated a greater apparent sensitivity in the KRAS^G12C^-mutant lines, whereas BrdU incorporation assays did not show a consistent relationship between KRAS mutation status and the IC₅₀ values for proliferation. The IC₅₀ values for BrdU incorporation were 22.2 µM for H358, 55.1 µM for CALU-1, 8.2 µM for A549, and 29.7 µM for H1299, without statistical difference between treatments (Fig. 1C).

Next, KRAS inhibition in the KRAS^G12C^-mutant cell lines (H358 and CALU-1) was associated with decreased AMD1 protein levels (Fig. 1D–E). In contrast, no consistent decrease in AMD1 was observed in A549 and H1299 cells, which lack the KRAS^G12C^ mutation accordingly with their reported insensitivity to adagrasib [20].

Finally, we assessed the combined effect of SAM486A and adagrasib across the four NSCLC cell lines using cell viability assays. According to the Loewe additivity model implemented in Combenefit, global synergy scores greater than + 10 indicate synergistic interactions, whereas values between − 10 and + 10 indicate additive effects [15]. Using this criterion, synergistic interactions were detected exclusively in KRAS^G12C^-mutant cells, as indicated by the blue-shaded synergy areas in the interaction maps (Fig. 1F). Global synergy scores were 18.06 for H358 cells and 18.43 for CALU-1 cells, whereas A549 and H1299 cells exhibited additive interactions, with global synergy scores of 7.33 and 0.25, respectively.

Collectively, these results suggest that the combination of SAM486A and adagrasib exerts a synergistic effect in vitro specifically in KRAS^G12C^-mutant tumor cells.

### SAM486 combined with adagrasib does not exhibit a synergistic effect in an orthotopic NSCLC model

**Fig. 2:**
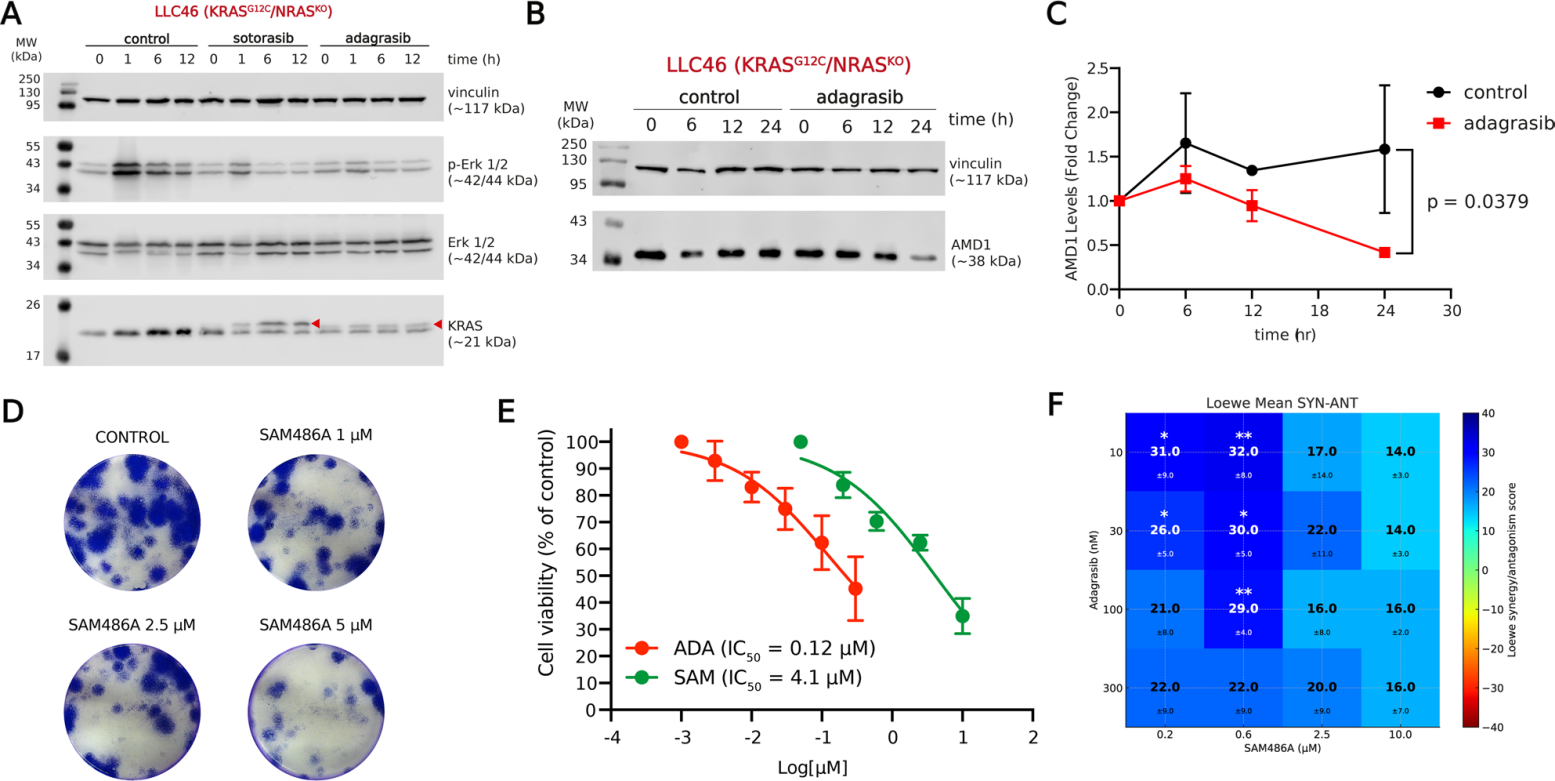
Validation of SAM486A and adagrasib effects on murine NSCLC KRAS^G12C^/NRAS^KO^ cells. **A** Effect of sotorasib (5 µM) and adagrasib (0.5 µM) on KRAS levels and ERK phosphorylation at 0, 1, 6, and 12 h of drug exposure. Red triangles indicate KRAS protein covalently bound to sotorasib and adagrasib inhibitors. The ERK1/2 panel corresponds to total ERK protein levels. **B** Representative blots showing the effect of adagrasib (0.5 µM) on AMD1 levels at 0, 6, 12 and 24 h. **C** Quantification of AMD1 levels in LLC46 cells after adagrasib exposure. The indicated *p*-value corresponds to the significant difference between treated and untreated groups over time, calculated using a two-way ANOVA (*n* = 3). **D** Representative images of colony growth in LLC46 cells after 5 days of SAM486A exposure. **E** Cell viability measured by the MTT assay after 72 h exposure to adagrasib and SAM486A. **F** Combination matrix for the adagrasib+SAM486A combination in vitro. Viability was measured at 72 h of treatment using MTT reduction. Results were analyzed using COMBENEFIT. Blue areas indicate synergistic interactions, while green tones represent additive effects. *: *p* < 0.05, compared with the Loewe theoretical model, calculated by t-test

 We used LLC46 (KRAS^G12C^/NRAS^KO^) cells as a validated syngeneic orthotopic model to assess the impact of the combination therapy on KRAS^G12C^-mutant cells [37]. Prior to conducting in vivo studies, we first validated the effect of the individual drugs and their combination in vitro (Fig. 2). Sotorasib was included as a reference KRAS^G12C^ inhibitor to functionally validate the responsiveness of the murine KRAS^G12C^ cell lines to direct KRAS inhibition, thereby serving as a pharmacological control confirming the presence and functional relevance of the KRAS^G12C^ mutation. In LLC46 cells, sotorasib and adagrasib reduced ERK1/2 phosphorylation (Fig. 2A), and adagrasib also lowered AMD1 levels after 24 h of exposure (Fig. 2B, C). Treatment with SAM486A significantly reduced colony formation (Fig. 2D). Cell viability decreased with both drugs, with an IC_50_ of 375 nM for adagrasib and 71 µM for SAM486A (Fig. 2E). Finally, the adagrasib + SAM486A combination exhibited strong synergy, with a combination score of 39.9 (Fig. 2F)

**Fig. 3:**
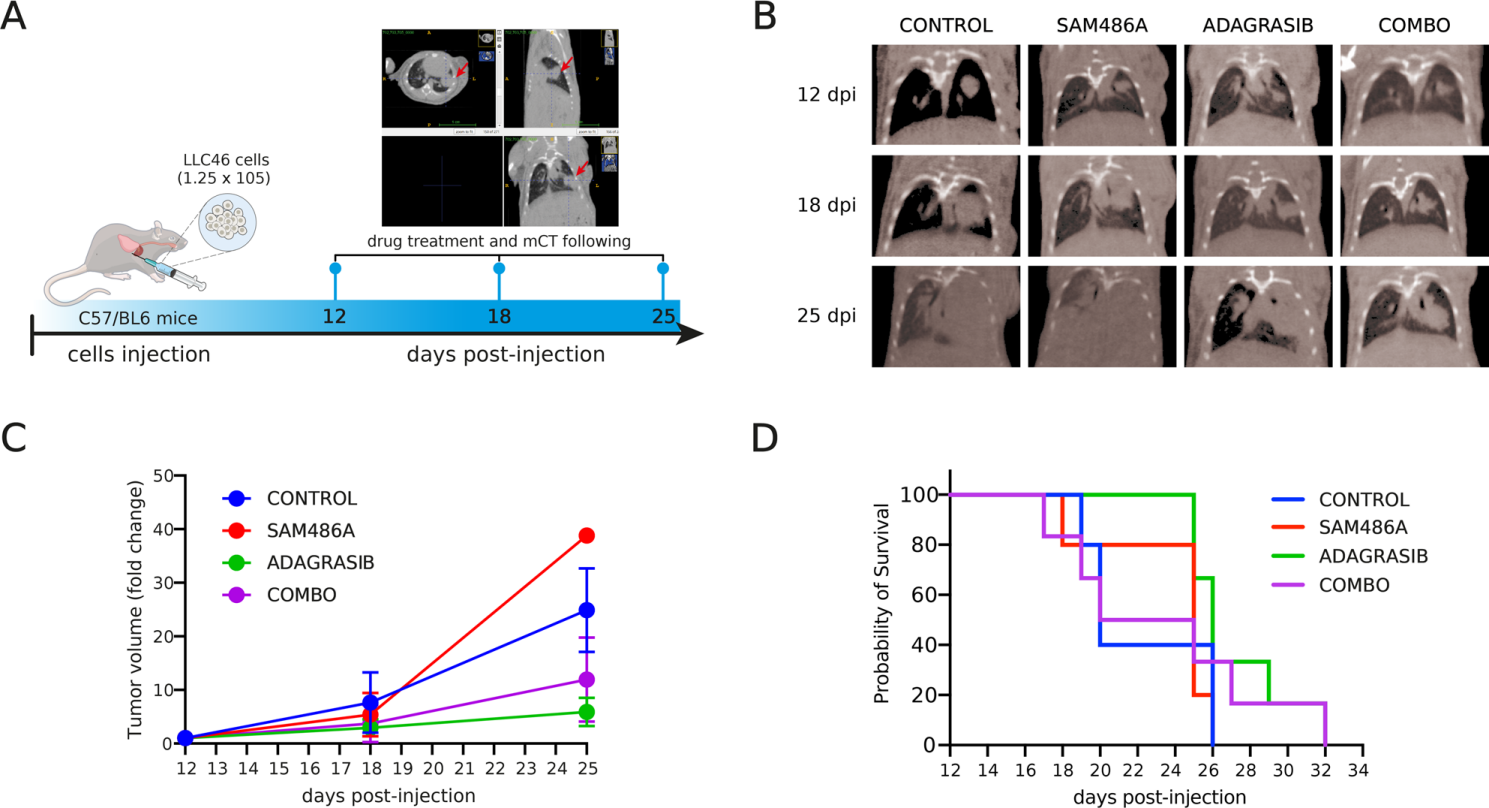
Effect of SAM486A and adagrasib combination therapy in an orthotopic NSCLC model. LLC46 (KRAS^G12C^/NRAS^KO^) cells were orthotopically implanted into the left lung of C57BL/6 mice and allowed to establish for 12 days before treatment and µCT scanning. **A** Schematic representation of the experimental timeline. **B** Representative µCT scans images of tumor-bearing mice. **C** Tumor volume growth curves for control, SAM486A, adagrasib, and combination (COMBO) cohorts (*n* = 5). **D** Survival curves comparing all four groups. For panels C and D, no significant differences were observed between groups

 Based on the synergistic activity of adagrasib and SAM486A in vitro, the drug combination was evaluated in an orthotopic lung tumor model, in which LLC46 cells were inoculated into the left lung of C57BL/6 mice (Fig. 3A and B).

Tumor growth was monitored over time using μCT scanning following treatment with adagrasib (30 mg/kg), SAM486A (30 mg/kg), or their combination (COMBO). In untreated mice, the average tumor volume was 175 mm³ at 12 days post-inoculation (p.i.), whereas treatment with adagrasib alone resulted in a tumor volume of 73 mm³. Similarly, treatment with the COMBO yielded a tumor volume of 86 mm³. SAM486A alone did not produce significant changes in tumor volume compared to the untreated group, which had a tumor volume of 160 mm³ (Fig. 3C). No statistically significant differences in tumor volume were observed among the treatment groups.

Additionally, no statistically significant improvement in survival was observed when comparing the COMBO to adagrasib alone. However, survival analysis revealed an increase in median survival from 8 days in the vehicle group to 14 days in the adagrasib-treated group and 13 days in the SAM486A-treated group. No further improvement was observed in the COMBO group, which had a median survival of 10.5 days (Fig. 3D)

### SAM486A combined with adagrasib have a synergistic effect in a metastatic model of NSCLC

 We selected the syngeneic tail vein injection model to assess treatment effects in a metastatic context. This approach allows us to study tumor cell colonization and metastatic behavior, particularly in the lungs. For this model, we used LL2 cells (Lewis Lung Carcinoma, KRAS^G12C^), which harbor both KRAS^G12C^ and NRAS^Q61H^ mutations [21]. These genetic alterations make LL2 cells a relevant model for studying tumors with diverse escape pathways to KRAS inhibition, providing insights into resistance mechanisms and therapeutic strategies [21]. Additionally, LL2 cells are well-documented for use in the tail vein injection model to investigate pulmonary tumor processes [13, 39, 43].

**Fig. 4:**
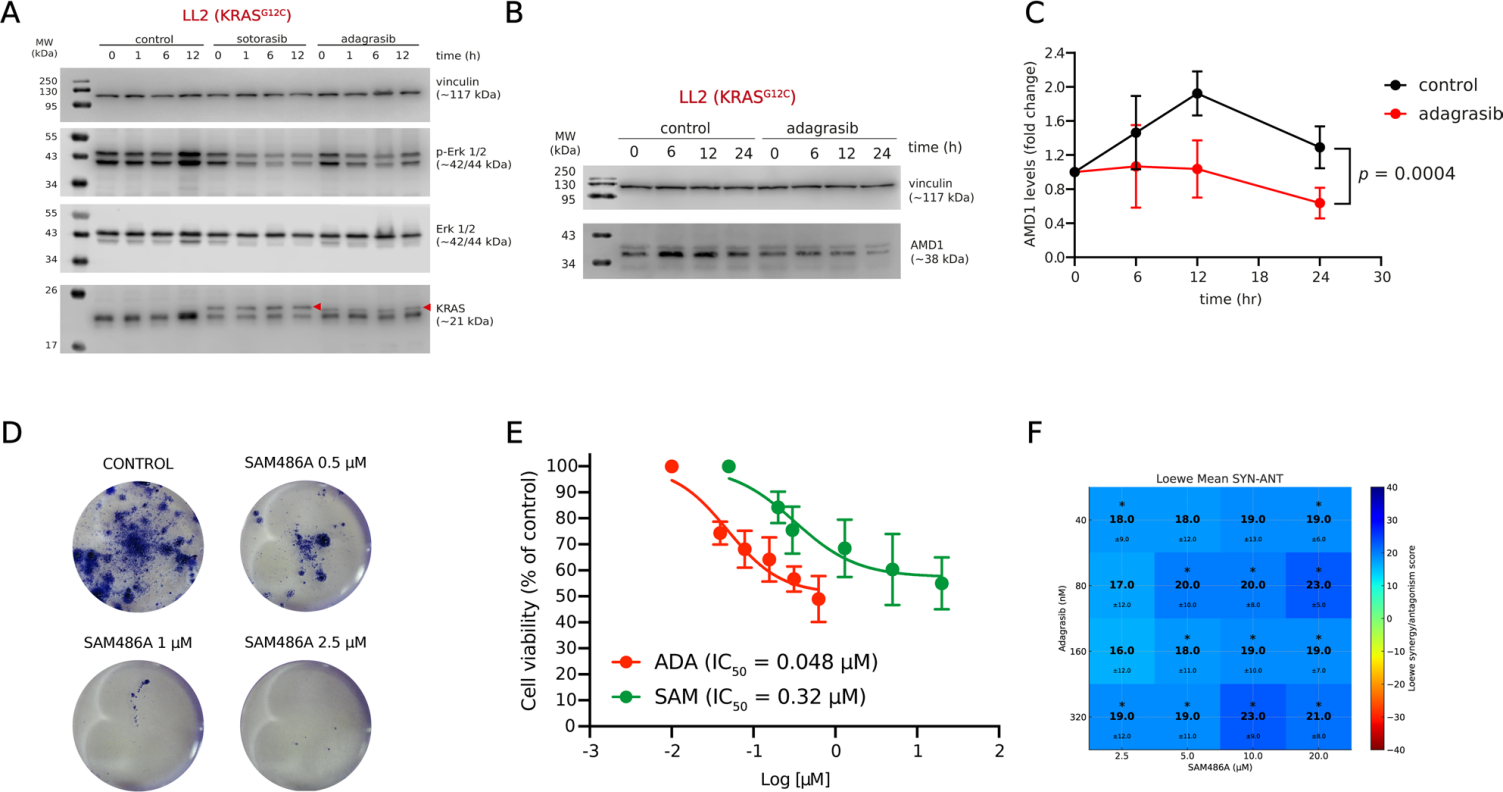
Validation of the effect of SAM486A and adagrasib (ADA) on murine NSCLC cells harboring the KRAS^G12C^ mutation. **A** Effect of sotorasib (5 µM) and adagrasib (0.5 µM) on RAS and ERK phosphorylation at 0, 1, 6, and 12 h of drug exposure. Red triangles correspond to RAS protein covalently bound to the sotorasib and adagrasib inhibitors. The ERK1/2 panel corresponds to total ERK protein levels. **B** Effect of adagrasib (0.5 µM) on AMD1 levels. **C** Quantification of AMD1 levels in LL2 cells after adagrasib exposure. The indicated *p*-value corresponds to the significant difference between treated and untreated groups over time, calculated using a two-way ANOVA (*n* = 3). **D** Representative images of colony growth in LL2 cells after 5 days of SAM486A exposure. **E**. Cell viability measured by MTT assay after treatment with SAM486A and adagrasib. **F** Combination matrix for the ADA + SAM combo in vitro. Viability was measured at 72 h of treatment using MTT reduction. Results were analyzed by COMBENEFIT. Blue areas show synergy points, while green tones indicate additive interaction. * = *p* < 0.05, compared with the Loewe theoretical model, calculated by t-test

 To characterize the response of LL2 cells, we exposed them to sotorasib and adagrasib. The appearance of a second band in the Western blot for RAS protein identification suggests that both drugs covalently bind to RAS, effectively reducing ERK1/2 phosphorylation (Fig. 4A). We further confirmed that adagrasib significantly reduces AMD1 levels in LL2 cells (Fig. 4B, C), while SAM486A significantly decreased cell proliferation (Fig. 4D). Pharmacologically, LL2 cells exhibited high sensitivity to adagrasib (EC_50_ = 48 nM) and greater sensitivity to SAM486A compared to the other cell lines studied (EC_50_ = 320 nM, Fig. 4E). Finally, adagrasib exhibited synergistic effects with SAM486A in vitro, with a synergy score of 22.69 (Fig. 4E).

**Fig. 5:**
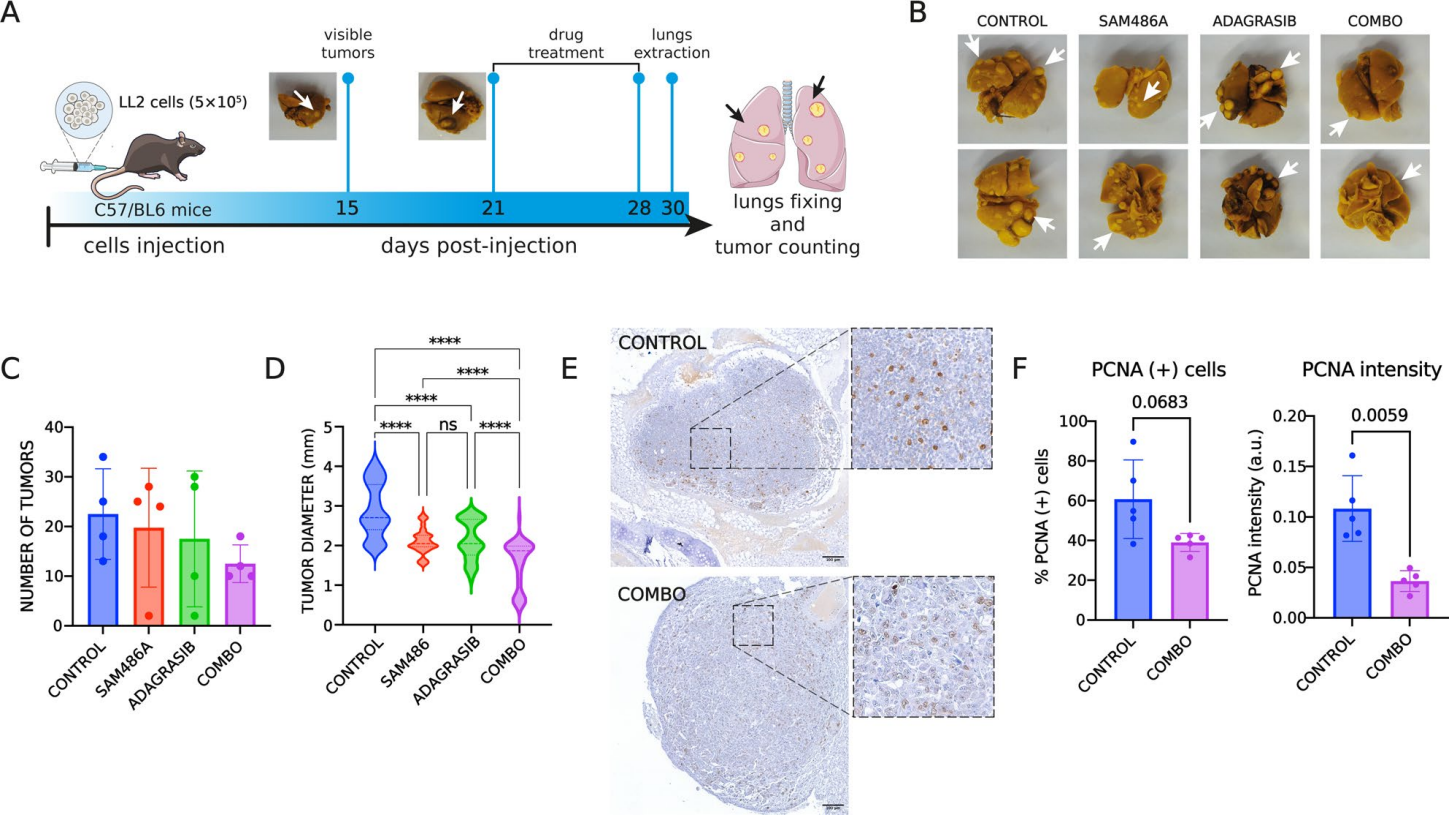
Effect of SAM486A and adagrasib combination therapy on tumor burden and proliferation in a metastatic NSCLC lung model. 500,000 LL2 cells were inoculated into the tail vein of C57BL/6 mice, and the combination (COMBO) of adagrasib (25 mg/kg/day) and SAM486A (25 mg/kg/day) was administered starting at 21 days post-inoculation for 7 days. **A** Diagram of the experimental design for the adagrasib (ADA) and SAM486A (SAM) combination study. **B** Representative lung images from control and treated groups. White arrows indicate visible tumors in each lung. **C** Effect of the adagrasib + SAM486A combination on the number of lung tumors per mouse in each treatment group (*n* = 4 mice per group). Data are shown as mean ± SD. No significant differences between groups were observed. **D** Tumor diameter distribution represented as violin plots, showing the full distribution of individual tumor measurements for each group. ****: *p* < 0.0001, determined by one-way ANOVA with Dunnett’s *post hoc* test, comparing the indicated groups. **E** Representative immunohistochemistry images showing PCNA levels in control and COMBO-treated (adagrasib + SAM486A) tumors. Dashed boxes indicate regions shown at higher magnification. **F** Quantification of PCNA immunohistochemistry showing the percentage of PCNA-positive cells and mean nuclear PCNA staining intensity in control and COMBO-treated tumors (a.u. : arbitrary units). Numbers above the bars indicate the corresponding *p*-values, calculated using an unpaired two-tailed *t*-test with Welch’s correction

We then performed in vivo experiments by injecting LL2 cells into the tail vein, leading to the development of visible tumors by day 21 post-inoculation (Fig. 5A). We observed a non-significant reduction in the number of tumors across groups: the control group averaged 22.5 tumors, compared to 19.75 tumors in the SAM486A-treated group, 17.5 tumors in the adagrasib-treated group and 12.5 tumors in the combination treatment group (Fig. 5B C). Additionally, there was a significant decrease in tumor diameter, with an average of 2.9 mm in the control group, 2.1 mm in the SAM486A-treated group, 2.2 mm in the adagrasib-treated group, and 1.6 mm in the COMBO-treated group, highlighting the enhanced effect of the combination therapy in reducing tumor size (Fig. 5D). Finally, we conducted immunohistochemistry (IHC) analysis for PCNA in tumor tissues, a crucial proliferation marker used to assess tumor biology and cancer progression [16, 41]. Tissue analysis revealed that the adagrasib + SAM486A combination (COMBO) reduced PCNA expression, indicating a decrease in tumor proliferation (Fig. 5E).

## Discussion

 Although KRAS-targeted drugs such as sotorasib and adagrasib represent promising therapeutic options for cancers harboring KRAS^G12C^ mutations, including lung, pancreatic, and colorectal cancers, these inhibitors face significant challenges due to the rapid emergence of resistance [30]. Documented mechanisms of resistance include secondary mutations in the KRAS gene, activation of alternative pathways upstream or downstream of KRAS, histological transformation (e.g., from adenocarcinoma to squamous cell carcinoma), and alterations within the tumor microenvironment [2]. Current strategies to overcome resistance involve combination therapies, such as dual blockade of upstream and downstream targets (e.g., EGFR and SHP2), as well as integration with standard chemotherapies (carboplatin, pemetrexed) or immune checkpoint inhibitors (pembrolizumab, atezolizumab) [5, 32].

 In this study, we demonstrate that adagrasib reduces AMD1 levels in KRAS^G12C^-mutant cells. This effect may sensitize KRAS^G12C^-harboring cells to concurrent inhibition of polyamine metabolism by SAM486A. AMD1 plays a central role in polyamine biosynthesis and has been implicated in the progression of hepatocellular carcinoma, prostate cancer, and breast cancer [18, 28, 36]. In prostate cancer, AMD1 overexpression is dependent on mTORC1 activity, and pharmacological inhibition of mTOR, but not MAPK, reduces active AMD1 levels [42, 45]. Given the interconnection between mTORC1 and MAPK signaling, a feedback loop may reinforce tumor growth. Targeting AMD1 could therefore disrupt this circuit and enhance therapeutic efficacy.

 We leveraged two murine lung cancer models (LL2 and its KRAS^G12C^/NRAS^KO^ derivative LLC46) to interrogate different therapeutic contexts. LL2 is a well-characterized NSCLC model with aggressive growth and high metastatic potential, making it particularly suitable for migration-focused studies such as the tail vein model [13, 39, 43]. In contrast, LLC46 [37] demonstrated markedly faster and more aggressive tumor growth in the orthotopic setting, which limited our ability to assess long-term combination effects in migration-based models.

 These biological differences likely explain the distinct responses observed in each system. In the orthotopic model, adagrasib monotherapy was highly effective, likely due to the absence of NRAS-mediated escape routes. As a result, the addition of SAM486A did not provide further benefit. Conversely, in the LL2 model, which is NRAS wild-type, the limited efficacy of adagrasib could be attributed to compensatory mechanisms driven by other RAS isoforms. For example, HRAS overexpression has been reported to support increased AMD1 activity under EGF stimulation [42]. In this context, SAM486A may suppress such alternative resistance pathways, thereby producing a synergistic effect. This finding is particularly relevant because LL2 preserves the full signaling complexity of tumor cells, potentially making it a more phenotypically realistic model. Observing synergy in this system thus strengthens the rationale for the combination strategy.

 The enhanced effect of SAM486A may also relate to processes not captured in the orthotopic model, such as cell migration and invasion. Indeed, AMD1 knockdown reduces migration in gastric cancer cells in vitro [42] and suppresses lung metastasis in hepatocellular carcinoma models in vivo [8]. Furthermore, the AMD1 inhibitor methylglyoxal-bis(cyclopentylamidinohydrazone) has been reported to prevent bone metastasis in a murine melanoma mode [40].

 Despite these promising findings, our study has limitations. First, while murine models provide valuable insights, they do not fully capture the heterogeneity of human tumors, which often display diverse genetic and phenotypic features, including epithelial-to-mesenchymal transition (EMT), a process closely linked to drug resistance and metastasis. Second, we did not extensively examine the tumor microenvironment or immune system contributions, both of which could critically influence treatment outcomes. This is particularly relevant since increased polyamine metabolism has been associated with immune evasion in lung adenocarcinoma [27]. Finally, although our data highlight the role of AMD1 and polyamine metabolism in KRAS^G12C^-driven tumors, the precise molecular mechanisms linking KRAS inhibition to AMD1 regulation remain to be elucidated. Addressing these questions will be essential to strengthen the translational potential of our findings.

 In conclusion, our study suggests that combining KRAS^G12C^ inhibitors with polyamine-targeting agents such as SAM486A could provide a novel therapeutic avenue to overcome resistance and improve outcomes in KRAS^G12C^-mutant cancers. Further research using more diverse models and mechanistic approaches will be necessary to validate the therapeutic potential of this strategy and to define the molecular interactions underlying its efficacy.

## Data Availability

All data generated or analyzed during this study are included in this published article. Additional datasets are available from the corresponding author upon reasonable request.
